# The effect of a web-based psychoeducation on emotional functioning, eating behaviors, and body image among premenopausal women with excess body weight

**DOI:** 10.1007/s00737-020-01077-1

**Published:** 2020-11-11

**Authors:** Kamila Czepczor-Bernat, Anna Brytek-Matera, Anna Staniszewska

**Affiliations:** 1 Institute of Psychology, University of Wroclaw, Wrocław, Poland; 2 Department of Experimental and Clinical Pharmacology, Medical University of Warsaw, Warsaw, Poland

**Keywords:** Web-based psychoeducation, eHealth, Premenopausal women, Excess body weight

## Abstract

The aims of this study were twofold: (1) to investigate the effectiveness of web-based psychoeducation for emotional functioning, eating behaviors, and body image among premenopausal women with excess body weight, and (2) to compare the efficacy of two types of web-based psychoeducation. Three hundred individuals were asked to volunteer in the present study. All participants were recruited in Poland from September 2017 to July 2019. Finally, a total of 129 premenopausal women took part in the research and signed informed consent. Their ages ranged between 18 and 48 years old (*M* = 32.28, *SD* = 7.65). Self-reported weight and height were recorded. BMI was calculated using self-reported data. Their average body mass index was 30.54 kg/m^2^ (*SD* = 3.69). In our randomized experiment, the participants were allocated into three groups: experimental group I (EG I, *N* = 43), experimental group II (EG II, *N* = 46), and wait list control group (CG, *N* = 40). Five questionnaires were included in the online survey at the baseline measurement (Day 0), at the end of psychoeducational intervention (Day 16) and 75 days from the start of the 15-day intervention (Day 76). Measurement tools included the Difficulties in Emotion Regulation Scale, the Positive and Negative Affect Schedule, the Mindful Eating Scale, the Three-Factor Eating Questionnaire, and the Body Attitude Test. Our eHealth web-based psychoeducation consisted of three modules: emotional functioning module (EG I: theoretically consistent approach (TCA) vs EG II: eclectic approach; EA), eating behaviors module (EG I, EG II: based on mindfulness-based eating training; MET), body image module (EG I, EG II: based on Cash’s prevention of body image disturbances; CPBID). The first experimental group (EG I) had intervention containing TCA, MET, and CPBID, while the second experimental group (EG II) EA, MET, and CPBID. According to between-group comparison, both types of web-based psychoeducation led to an increase in adaptive emotion regulation (Day 16: EG I vs CG: *p* < 0.001, EG II vs CG: *p* < 0.001; Day 76: EG I vs CG: *p* < 0.01, EG II vs CG: *p* < 0.001). In EG I, the intervention resulted in a higher reduction (than in CG) in emotional eating (Day 16: *p* < 0.01, Day 76: *p* < 0.01), uncontrolled eating (Day 16: *p* < 0.05, Day 76: *p* < 0.05), and negative appreciation of body size (Day 16: *p* < 0.01, Day 76: *p* < 0.01). In EG II, a lower level of emotional eating was found on Day 76 (EG II vs CG: *p* < 0.05). Two months after completion of the 15-day intervention, no statistically significant reduction for BMI was observed in either experimental group (*p* > 0.05). The effectiveness of both types of web-based psychoeducation was also confirmed in within-group comparison (Day 0 vs Day 16 and Day 0 vs Day 76). There was a significant increase in emotion regulation and mindful eating, as well as a decrease in emotional eating, uncontrolled eating, negative appreciation of body size, lack of familiarity with one’s body, and the experiencing of negative emotions in both experimental groups (EG I, EG II). Both types of web-based psychoeducation might have to be considered in creating future web-based psychoeducation among premenopausal women with excess body weight.

## Introduction

Many electronic health (eHealth) interventions have been assessed among physically and mentally ill patients (e.g., Dening et al. 2019; Gao et al. 2019). The American Psychological Association (2015) suggests that web-based interventions have become a popular and acceptable method of support and therapy around the world and the effectiveness of many of them has been presented in numerous publications (e.g., Jahangiry et al. 2017; Ploeg et al. 2018). The high prevalence of computer use provides novel possibilities for enhancing health service delivery (American Psychological Association 2015; Dening et al. 2019). Therefore, eHealth interventions have also been used in the treatment of patients with obesity and their effectiveness has been confirmed from meta-analyses (e.g., Hutchesson et al. 2015). However, most focused only on body weight (Hutchesson et al. 2015).

The World Health Organization (WHO) and the European Association for the Study of Obesity still recommend seeking new ways to help patients and developing new interventions to be more effective and more suited to their needs (McGowan 2016; World Health Organization 2014; Yumuk et al. 2014). These recommendations were related to the fact that: (a) the spread of obesity was constantly increasing and the cost of treatment constantly rising, (b) some of the studies have indicated that psychological factors may be involved in the development, maintenance, and treatment of obesity (e.g., Castelnuovo et al. 2016; Hemmingsson 2014; Marks 2015; McGowan 2016; Raman et al. 2013; Spieker and Pyzocha 2016; World Health Organization 2014; Yumuk et al. 2014). A pertinent question is therefore whether traditional approaches to obesity treatment may be supplemented by web-based psychoeducation that could be used by interdisciplinary teams in the future.

Obesity has become one of the major public health problems (Ng et al. 2013). Interestingly, it was reported that there were many countries in the world (e.g., Poland) in which the prevalence of obesity above 25% was present only among women (WHO 2014). Moreover, the Central Statistical Office (2006, 2011, 2016) data indicated that the number of overweight women has doubled from 1996 to 2014. Therefore, the most important task at present is to stop the increase in the prevalence of this major public health problem by developing and implementing new treatment methods among overweight and obese Polish women.

There is a very large body of literature on the treatment of patients with obesity (e.g., Hutchesson et al. 2015; Jahangiry et al. 2017; Kirk et al. 2012; McGowan 2016; Yumuk et al. 2014). However, specialists still point to important problems (Kirk et al. 2012). According to healthcare professionals, one of the barriers to obesity care and treatment has been the patient’s poor emotional and mental health (Kaplan et al. 2018). Patients believe that the difficulty arises from a lack of knowledge and motivation to change, an inability to control eating behaviors, and a preference for unhealthy food (Kaplan et al. 2018). The significance of these variables in the development and maintenance of obesity was confirmed by the models developed by Marks (2015), Hemmingsson (2014), and Raman et al. (2013). These models indicated that maladaptive emotional functioning was associated with unhealthy eating behaviors and distorted body image.

The need to implement new interventions was also indicated by researchers dealing with the use of mindful eating training in the treatment of obesity (Kristeller and Hallett 1999; Kristeller and Wolever 2011; Wnuk and Du 2017). These researchers postulated that in addition to changing abnormal eating behaviors, one should focus on changing one’s emotional functioning and the relationship with one’s own body (Kristeller and Hallett 1999; Kristeller and Wolever 2011; Wnuk and Du 2017). Interestingly, one of these studies suggested that to achieve this goal, specialists should combine mindful eating training with Emotional Schema Therapy (Wnuk and Du 2017). The efficacy of Emotional Schema Therapy is still being verified among overweight and obese patients. In turn, mindful eating training has often been used as a basic method of treatment among patients with excess body weight (e.g., Kristeller and Wolever 2011; Morvaridi et al. 2018; Seguias and Tapper 2018; Schultz 2017; Washington et al. 2017; Wnuk and Du 2017). It turned out that intervention in the field of mindfulness promoted (both among overweight and obese people) (a) a reduction in anxiety and depression (Daubenmier et al. 2011; Kristeller et al. 2013); (b) the development of a proper attitude towards food and its enjoyment (Smart et al. 2015); (c) the regulation of problematic nutrition (Ouwens et al. 2015; Katterman et al. 2014; O’Reilly et al. 2014); (d) better control of food intake accompanied by an increased awareness of hunger and satiety signals (Kristeller and Hallett 1999); (e) an improvement in the assessment of one’s body (Bush et al. 2014); and (f) an increase in weight loss effectiveness (Kidd et al. 2013).

To the best of our knowledge: (a) the above suggestion (combine mindful eating training with Emotional Schema Therapy) has not been verified in studies, therefore, it would be necessary to verify whether the co-occurrence of Emotional Schema Therapy and mindful eating training results in improved emotional functioning, eating behaviors, and body image (Wnuk and Du 2017); (b) the effectiveness of web-based psychoeducation (aimed equally at improving emotional functioning, eating behaviors, and body image) has not yet been verified among overweight and obese people (especially among Polish women).

A pertinent research question is therefore whether web-based psychoeducation can also be used to enhance emotional functioning, eating behaviors, and body image among Polish women by providing a new psycho-educational, three-module intervention. The aims of this study were twofold: (1) to investigate the effectiveness of web-based psychoeducation for emotional functioning, eating behaviors, and body image among premenopausal women with excess body weight, and (2) to compare the efficacy of two types of web-based interventions. It was hypothesized that the implementation of both types of web-based psychoeducation would result in improved emotional functioning, eating behaviors, and body image. However, based on previous research into emotional functioning, it was hypothesized that a theoretically consistent approach (TCA; an approach based on Emotional Schema Therapy) would be more effective than an eclectic approach (EA; the approach consisted of a combination of different approaches to treating emotional functioning, e.g., a combination of compassion-focused therapy (Gilbert 2009) and dialectical behavioral therapy (Linehan 2014)).

## Materials and methods

### Participants

**Fig. 1:**
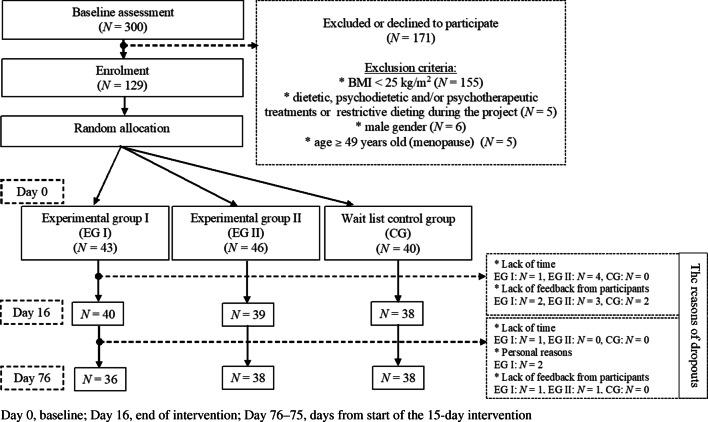
Flow diagram of participants throughout the study

A total of 300 women and men were assessed who were interested in participating in the study. Next, only 129 women who fulfilled the inclusion criteria ((a) BMI ≥ 25 kg/m^2^; (b) no other interventions during the project; (c) female gender; (d) age ≤ 48 years) were enrolled in the study and invited to participate in the web-based psychoeducation (Fig. 1). Other participants (*N* = 171) were deemed to be ineligible and were informed of this fact. Written informed consent forms were obtained before the participants were enrolled in the study.

In our randomized experiment, 129 women were allocated into three groups: experimental group I (EG I), experimental group II (EG II), and wait list control group (CG). Self-reported weight and height were recorded. Body mass index (BMI) was calculated using self-reported data.

Details on sample size calculations and drop outs can be found here.

Details on exclusion criteria can be found here.

### Measures

Five questionnaires were included in the online survey at the baseline measurement (Day 0), at the end of the psychoeducational intervention (Day 16), and 75 days from start of the 15-day intervention (Day 76). Moreover, sociodemographic variables (e.g., body weight, BMI) were assessed at Day 0 and Day 76. Initially, we used a standard forward-backward translation procedure (it included two questionnaires which did not have the Polish adaptation—the Difficulties in Emotion Regulation Scale, the Mindful Eating Scale). The Positive and Negative Affect Schedule, the Three-Factor Eating Questionnaire, and the Body Attitude Test had Polish adaptation. We obtained permission to use all questionnaires from their authors. Regarding the Positive and Negative Affect Schedule, the licenses for its use were purchased in the Psychological Test Laboratory of the Polish Psychological Association.

#### The Difficulties in Emotion Regulation Scale

The questionnaire consisted of 36 items and included a general scale and six subscales related to the process of regulating emotions: (a) non-acceptance of emotional responses (example item: “When I’m upset, I feel guilty for feeling that way”); (b) difficulty engaging in goal-directed behavior (example item: “When I’m upset, I have difficulty concentrating”); (c) impulse control difficulties (example item: “When I’m upset, I lose control over my behavior”); (d) lack of emotional awareness (example item: “I am attentive to my feelings”); (e) limited access to emotion regulation strategies (example item: “When I’m upset, I believe that I’ll end up feeling very depressed”); and (f) lack of emotional clarity (example item: “I have difficulty making sense of my feelings”) (Gratz and Roemer 2004). Participants responded on a 5-point scale (from almost never to almost always). The lower the score was, the higher the intensity of adaptive emotional regulation. The Difficulties in Emotion Regulation Scale has demonstrated adequate construct as well as predictive validity (Gratz and Roemer 2004). Cronbach’s alpha coefficient (reliability) for the general scale was 0.88 and for individual subscales: *α*_non-acceptance of emotional responses_ = 0.85, *α*_difficulties engaging in goal-directed_ = 0.89, *α*_impulse control difficulties_ = 0.86, *α*_lack of emotional awareness_ = 0.80, *α*_limited access to emotion regulation strategies_ = 0.88, *α*_lack of emotional clarity_ = 0.84 (Gratz and Roemer 2004). Our analysis focused solely on the general scale (*α* = 0.95).

#### The Positive and Negative Affect Schedule

The questionnaire was used to measure the intensity of emotions (Brzozowski 2010; Watson et al. 1998). Version S20 included 10 items related to the strength of experiencing negative emotions (subscale: NU-10—negative affective states; example of feeling: “Guilty”) and 10 items related to the strength of experiencing positive emotions (subscale: PU-10—positive affective states; example of the feeling: “Enthusiastic”). Participants responded on a 5-point scale (from not at all to very much). Higher scores indicated a more intense feeling or emotion. Cronbach’s alpha coefficient (reliability) for subscales in Brzozowski’s study (2010) ranged from 0.73 to 0.95 and these scales had good validity. The presented analysis only focused on the negative emotions scale (*α* = 0.94).

#### The Mindful Eating Scale

The questionnaire involved 28 items and included a general scale and six subscales relating to mindful eating: (a) acceptance (example item: “I tell myself I shouldn’t be hungry”); (b) awareness (example item: “I notice how my food looks”); (c) non-reactivity (example item: “I need to eat like clockwork”); (d) routine (example item: “I eat the same thing for lunch each day”); (e) distractibility (example item: “I eat something without really being aware of it”); and (f) unstructured (example item: “I eat at my desk or computer”) (Hulbert-Williams et al. 2014). Participants responded on a 4-point scale (from never to usually). The higher the score they got, the higher the level of mindful eating was. The Mindful Eating Scale has demonstrated adequate validity (Hulbert-Williams et al. 2014). Cronbach’s alpha coefficient (reliability) for the general scale was 0.86 and for individual subscales: *α*_acceptance_ = 0.89, *α*_awareness_ = 0.82, *α*_non-reactivity_ = 0.77, *α*_routine_ = 0.75, *α*_distractibility_ = 0.81, *α*_unstructured_ = 0.60 (Hulbert-Williams et al. 2014). The presented analysis only focused on the general scale (*α* = 0.84).

#### The Three-Factor Eating Questionnaire

The Three-Factor Eating Questionnaire (TFEQ-R18) (Brytek-Matera et al. 2017; Karlsson et al. 2010) contained three subscales: (a) restrictive eating (example item: “I consciously hold back at meals in order not to weight gain”); (b) uncontrolled eating (example item: “Sometimes when I start eating, I just can’t seem to stop”); and (c) emotional eating (example item: “When I feel blue, I often overeat”). The results of the subjects were finally recoded on a 4-point scale. The higher the score, the higher the maladaptive eating behaviors were. The Three-Factor Eating Questionnaire-R18 had good validity (Karlsson et al. 2010). Cronbach’s alpha coefficient (reliability) for subscales in the Polish adaptation of the questionnaire was *α*_restrictive eating_ = 0.78, *α*_uncontrolled eating_ = 0.84, and *α*_emotional eating_ = 0.86 (Brytek-Matera et al. 2017). The presented analysis only focused on the subscales: *α*_uncontrolled eating_ = 0.84 and *α*_emotional eating_ = 0.88.

#### The Body Attitude Test

The questionnaire was used to measure attitudes towards the body (Brytek-Matera and Probst 2014; Probst et al. 1995). It included 20 items and was composed of three subscales: (a) negative appreciation of body size (example item: “I think I’m too thick”); (b) lack of familiarity with one’s body (example item: “It’s easy for me to relax physically”); (c) general dissatisfaction (example item: “When I look at myself in the mirror, I’m dissatisfied with my own body”). Participants responded on a 5-point scale (from never to always). The higher the sum of scores, the worse the functioning in the context of body image. The Body Attitude Test has demonstrated adequate validity (Probst et al. 1995). Cronbach’s alpha coefficient (reliability) for subscales in the non-clinical population was *α*_negative appreciation of body size_ = 0.80, *α*_lack of familiarity with body_ = 0.68, and *α*_general dissatisfaction_ = 0.80 (Brytek-Matera and Probst 2014). The presented analysis only focused on the two subscales—negative appreciation of body size (*α* = 0.80) and lack of familiarity with one’s body (*α* = 0.85).

#### Sociodemographic questionnaire

The data set contained information on age, sex, height, weight, and both physical and mental illnesses.

### Variables

The between-subjects independent variable was the web-based psychoeducation in the three experimental conditions (EG I—first experimental group I, EG II—second experimental group II, CG—control group). The within-subjects independent variable was the time of measurements in the study (Day 0—baseline, Day 16—end of psychoeducational intervention, and Day 76–75—days from start of the 15-day intervention).

The dependent variables were (1) emotional functioning: emotion regulation, negative emotions; (2) eating behaviors: mindful eating, emotional eating, uncontrolled eating; (3) body image: negative appreciation of body size, lack of familiarity with body; (4) body mass index (BMI); and (5) body weight (kg).

### Procedure

**Fig. 2:**
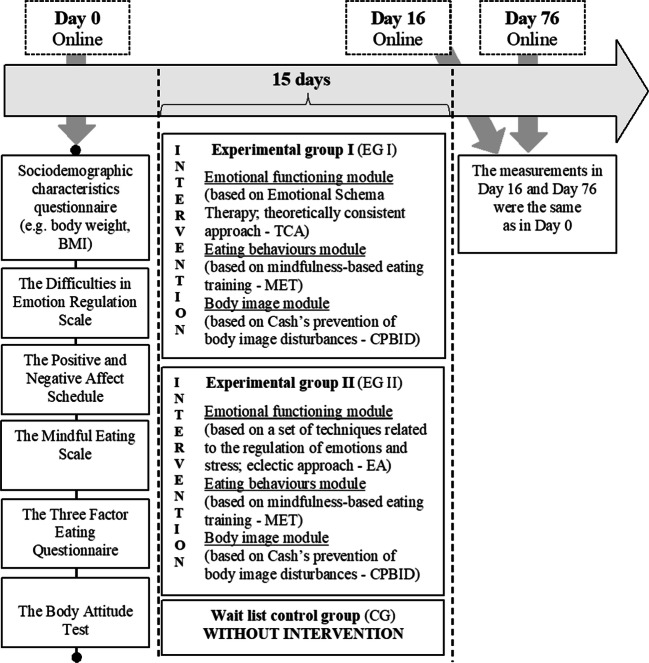
Measurements and web-based psychoeducation. Note*.* More details for the 2-month follow-up can be found here

The participants were recruited using convenience sampling. All participants were recruited in Poland via flyers (e.g., obesity treatment centers) and via social media networks (e.g., Facebook fan pages about nutrition) from September 2017 to July 2019. Initially, respondents received a link to a survey in which they answered questions related to the exclusion criteria. They also provided an e-mail address to which respondents (who were selected using exclusion criteria; Fig. 1) received a message inviting them to take part in further stages of the study and information about their new project’s anonymous e-mail address ((except the researchers conducting this study) nobody could identify the participants from this e-mail address). In the next stages of the study, participants used only the project’s e-mail address—it was to this new e-mail address that participants were sent subsequent measurements and intervention materials. A detailed diagram of the study is presented in Fig. 2.

Our eHealth web-based psychoeducation consisted of three modules (Fig. 2): emotional functioning module (EG I: theoretically consistent approach, TCA vs EG II: eclectic approach, EA), eating behaviors module (EG I, EG II: based on mindfulness-based eating training; MET), and body image module (EG I, EG II: based on Cash’s prevention of body image disturbances; CPBID).

The first experimental group (EG I) had an intervention containing TCA, MET, and CPBID and the second experimental group (EG II) EA, MET, and CPBID. Thus, the experimental groups differed only in the emotional functioning module. In experimental group I (EG I), the theoretically consistent approach was based on Emotional Schema Therapy (Leahy et al. 2014), while in experimental group II (EG II), the eclectic approach consisted of a combination of different approaches to treating emotional functioning, e.g., a combination of compassion-focused therapy (Gilbert 2009), acceptance and commitment therapy (ACT; Hayes et al. 2013), and dialectical behavioral therapy (Linehan 2014).

In the theoretically consistent approach based on Emotional Schema Therapy (Leahy et al. 2014), we used several exercises, e.g., “Acceptance of Emotions.” This exercise develops the ability to adopt an accepting attitude towards emerging feelings and train an adaptive strategy of coping with emotions based on being in touch with them when they emerge and at the same time eliminating non-adaptive strategies. For this purpose, the form 2.9 “How to Accept Difficult Feelings” was used (participants developed methods that they could use in emotionally difficult situations; exemplary questions/guidance: “What is the feeling that is hard to accept?,” “What does it mean to you if you accepted that you had this feeling?,” “What are the advantages and disadvantages of accepting the feeling?,” “Shift attention to other activities and things around you.,” “Are there productive, rewarding, or pleasurable things to do?”; Leahy et al. 2014, pp. 297).

In this eclectic approach, based on a combination of different approaches to treating emotional functioning, several exercises have been used, e.g., “Monsters on the Bus.” It develops the ability to adopt an accepting attitude towards emerging feelings, make people more aware of the universality of experienced emotions/ambivalent states, and increase their ability to manage emotions and accept them in everyday life. For this purpose, the ACT metaphor described in form 6.3 was used (for a detailed record of the metaphor, see Leahy et al. 2014, pp. 184–185).

In both groups (EG I, EG II), the aim was to achieve the same goal in emotional functioning but using different approaches (e.g., aim: increased level of acceptance of emotions—EG I: Emotional Schema Therapy techniques, EG II: acceptance and commitment therapy techniques; aim: increased level of awareness and tolerance of mixed and/or ambivalent emotions—EG I: Emotional Schema Therapy techniques, EG II: emotion-focused therapy techniques). The modules relating to eating behaviors (MET) and body image (CPBID) were the same in EG I and EG II. Details on our web-based psychoeducation can be found here.

Participants were not offered any remuneration. The research was approved by the Research Ethics Committee (no. 01/E/10/2017). All participants were treated in strict compliance with the Helsinki Declaration (2001). It is worth adding that all women were informed that participation in the study was voluntary and that, at any stage, they were free to withdraw without any consequences. Respondents were assured that they would remain anonymous, and that participation in the research would not involve any financial benefits.

### Data analysis

A mixed-design ANOVA was conducted using the Statistical Package for the Social Sciences version 22. The *p* value was less than 0.05. Bonferroni correction was applied in the post hoc multiple comparisons (between-group comparison and within-group comparison) (Field 2018; Niewiarowski et al. 2013). To evaluate the effect size, the criteria were used (Miles and Shevlin 2001): 0.01–0.06, small effect; 0.06–0.14, medium effect; above 0.14, large effect.

## Results

### Descriptive statistics

Table 1 presents the baseline characteristics for premenopausal women with excess body weight. The results presented in Table 1 show that the groups differ significantly only in the incidence of overweight women in each group. In terms of other baseline characteristics of the group, they are similar.

**Table 1:** Baseline characteristics of the participants (Day 0)

Groups	Experimental group I (EG I)	Experimental group II (EG II)	Wait list control group (CG)	
Variables				
	Sociodemographic characteristic *N* (%)			Chi-square test
Body mass index (kg/m^2^)				
Overweight	28 (65.12)	24 (52.17)	11 (27.50)	***χ***^**2**^ **(1,** ***N*** **= 63) = 7.52;** ***p*** **< 0.05**
Obesity	15 (34.88)	22 (47.83)	29 (72.50)	*χ*^2^ (1, *N* = 66) = 4.46; *p* > 0.05
	*M* (SD)			One-way ANOVA
Age (years)	32.67 (8.25)	33.00 (6.91)	31.03 (7.84)	*F*(2, 126) = 0.77; *p* > 0.05
Weight (kg)	84.27(15.37)	85.88 (11.86)	89.23 (9.67)	*F*(2, 126) = 1.67; *p* > 0.05
Height (cm)	168.37 (7.34)	167.54 (4.78)	168.20 (6.70)	*F*(2, 126) = 0.21; *p* > 0.05
Body mass index (kg/m^2^)	29.61 (4.17)	30.59 (3.97)	31.50 (2.43)	*F*(2, 126) = 2.79; *p* > 0.05
	Clinical characteristic *N* (%)			Chi-square test
Mental and physical illnesses^a^				
Yes	7 (16.28)	11 (23.91)	3 (7.50)	*χ*^2^ (1, *N* = 21) = 4.57; *p* > 0.05
No	36 (83.72)	35 (76.09)	37 (92.50)	*χ*^2^ (1, *N* = 108) = 0.06; *p* > 0.05

The statistically significant outcomes were highlighted in bold

^a^EG I: Hashimoto’s thyroiditis (*N* = 1), spine diseases (*N* = 1), insulin resistance (*N* = 1), hyperthyroidism (*N* = 1), polycystic ovary syndrome (*N* = 1), depression (*N* = 2); EG II: Lyme disease (*N* = 1), spine diseases (*N* = 2), joint disease (*N* = 1), Hashimoto’s thyroiditis (*N* = 1), hypothyroidism (*N* = 4), depression (*N* = 2); CG: Hashimoto’s thyroiditis (*N* = 1), joint disease (*N* = 1), depression (*N* = 1)

Table 2 presents the descriptive statistics for premenopausal women with excess body weight.

**Table 2:** Descriptive statistic

Stage	Day 0	Day 16	Day 76
Dependent variable			
*M* (*SD*)			
Emotional functioning			
Emotion regulation	EG I: 98.70 (28.35)	EG I: 73.00 (13.48)	EG I: 72.47 (12.10)
	EG II: 97.63 (27.85)	EG II: 75.87 (21.39)	EG II: 75.61 (17.76)
	CG: 99.83 (26.69)	CG: 99.18 (26.08)	CG: 99.53 (26.05)
Negative emotions	EG I: 21.02 (9.29)	EG I: 17.60 (7.78)	EG I: 17.03 (6.86)
	EG II: 22.43 (9.89)	EG II: 19.36 (6.82)	EG II: 18.87 (6.27)
	CG: 22.28 (9.07)	CG: 20.97 (8.83)	CG: 20.87 (7.91)
Eating behaviors			
Mindful eating	EG I: 72.81 (11.77)	EG I: 76.70 (12.41)	EG I: 75.89 (11.49)
	EG II: 70.87 (11.63)	EG II: 73.92 (12.10)	EG II: 74.92 (11.86)
	CG: 69.40 (10.40)	CG: 69.97 (10.69)	CG: 69.68 (10.78)
Emotional eating	EG I: 8.95 (3.12)	EG I: 6.78 (2.51)	EG I: 6.64 (2.54)
	EG II: 8.43 (3.15)	EG II: 7.31 (2.58)	EG II: 6.89 (2.66)
	CG: 8.80 (2.73)	CG: 8.66 (2.76)	CG: 8.55 (2.62)
Uncontrolled eating	EG I: 20.79 (6.80)	EG I: 18.65 (6.02)	EG I: 18.69 (6.30)
	EG II: 22.74 (6.38)	EG II: 20.36 (5.55)	EG II: 19.95 (5.31)
	CG: 23.13 (6.96)	CG: 22.82 (7.00)	CG: 22.68 (7.01)
Body image			
Negative appreciation of body size	EG I: 22.05 (7.98)	EG I: 17.28 (7.05)	EG I: 17.14 (7.21)
	EG II: 22.39 (7.31)	EG II: 19.56 (7.70)	EG II: 19.34 (7.94)
	CG: 22.93 (6.80)	CG: 22.37 (7.15)	CG: 22.74 (7.02)
Lack of familiarity with the body	EG I: 14.42 (8.21)	EG I: 12.55 (7.33)	EG I: 11.94 (7.48)
	EG II: 15.87 (7.47)	EG II: 14.31 (6.91)	EG II: 14.08 (6.51)
	CG: 15.85 (7.99)	CG: 15.63 (8.11)	CG: 15.82 (7.93)
BMI (kg/m^2^)	EG I: 29.61 (4.17)		EG I: 29.59 (4.49)
	EG II: 30.59 (3.97)		EG II: 30.41 (4.11)
	CG: 31.50 (2.43)		CG: 31.01 (2.65)
Body weight (kg)	EG I: 84.27 (15.37)		EG I: 84.30 (16.20)
	EG II: 85.88 (11.86)		EG II: 85.16 (12.41)
	CG: 89.23 (9.67)		CG: 88.00 (10.32)

### Evaluation of a web-based psychoeducation: mixed-design ANOVA

The results of mixed-design ANOVA are presented in Table 3.

**Table 3:** Main effect of condition (C), main effect of time (T), and interaction effect of the condition and time (I)

Variable	Effect	
Emotional functioning		
Emotion regulation	C	***F*(2, 109) = 9.67;** ***p*** **< 0.001;** ***η***_**p**_^**2**^ **= 0.151**
	T	***F*(2, 218) = 76.92;** ***p*** **< 0.001;** ***η***_**p**_^**2**^ **= 0.414**
	I	***F*(4, 218) = 20.48;** ***p*** **< 0.001;** ***η***_**p**_^**2**^ **= 0.273**
Negative emotions	C	*F*(2, 109) = 1.46; *p* > 0.05; *η*_p_^2^ = 0.026
	T	***F*(2, 218) = 22.64;** ***p*** **< 0.001;** ***η***_**p**_^**2**^ **= 0.172**
	I	*F*(4, 218) = 2.44; *p* > 0.05; *η*_p_^2^ = 0.043
Eating behaviors		
Mindful eating	C	*F*(2, 109) = 2.26; *p* > 0.05; *η*_p_^2^ = 0.040
	T	***F*(2, 218) = 17.87;** ***p*** **< 0.001;** ***η***_**p**_^**2**^ **= 0.141**
	I	***F*(4, 218) = 3.48;** ***p*** **< 0.05;** ***η***_**p**_^**2**^ **= 0.060**
Emotional eating	C	*F*(2, 109) = 3.01; *p >* 0.05; *η*_p_^2^ = 0.052
	T	***F*(2, 218) = 39.15;** ***p*** **< 0.001;** ***η***_**p**_^**2**^ **= 0.264**
	I	***F*(4, 218) = 12.03;** ***p*** **< 0.001;** ***η***_**p**_^**2**^ **= 0.181**
Uncontrolled eating	C	*F*(2, 109) = 2.79; *p* > 0.05; *η*_p_^2^ = 0.049
	T	***F*(2, 218) = 22.86;** ***p*** **< 0.001;** ***η***_**p**_^**2**^ **= 0.173**
	I	***F*** **(4, 218) = 4.13;** ***p*** **< 0.05;** ***η***_**p**_^**2**^ **= 0.070**
Body image		
Negative appreciation of body size	C	*F*(2, 109) = 3.10; *p* > 0.05; *η*_p_^2^ = 0.054
	T	***F*(2, 218) = 38.79;** ***p*** **< 0.001;** ***η***_**p**_^**2**^ **= 0.262**
	I	***F*(4, 218) = 8.86;** ***p*** **< 0.001;** ***η***_**p**_^**2**^ **= 0.140**
Lack of familiarity with body	C	*F*(2, 109) = 1.52; *p* > 0.05; *η*_p_^2^ = 0.027
	T	***F*(2, 218) = 12.17;** ***p*** **< 0.001;** ***η***_**p**_^**2**^ **= 0.100**
	I	***F*(4, 218) = 3.59;** ***p*** **< 0.05;** ***η***_**p**_^**2**^ **= 0.062**
Body mass index		
BMI (kg/m^2^)	C	*F*(2, 109) = 1.52; *p* > 0.05; *η*_p_^2^ = 0.027
	T	***F*(1, 109) = 7.57;** ***p*** **< 0.01;** ***η***_**p**_^**2**^ **= 0.065**
	I	*F*(2, 109) = 2.80; *p* > 0.05; *η*_p_^2^ = 0.023
Body weight		
Body weight (kg)	C	*F*(2, 109) = 0.99; *p* > 0.05; *η*_p_^2^ = 0.018
	T	***F*(1, 109) = 8.77;** ***p*** **< 0.01;** ***η***_**p**_^**2**^ **= 0.074**
	I	*F*(2, 109) = 1.20; *p* > 0.05; *η*_p_^2^ = 0.022

Greenhouse-Geisser epsilon correction was 0.603, 0.857, 0.663, 0,792, 0.834, 0.662, and 0.570. It should be remembered that BMI and body weight were measured twice, which results in a lack of sphericity assessment (Field 2018). The statistically significant outcomes were highlighted in bold. Details on the effect size can be found here

According to between-group comparisons, both types of web-based psychoeducation led to an increase in adaptive emotion regulation and the results of the experimental groups (EG I, EG II) differed from the control group (CG) on Day 16 and Day 76. In EG I, the intervention resulted in a higher reduction (than in CG) in emotional eating, uncontrolled eating, and negative appreciation of body size on Day 16 and Day 76. Interestingly, the second type of intervention (EG II) also contributed to a decrease in the level of emotional eating and the results of EG II differed significantly from CG on Day 76. No significant differences were observed in relation to other dependent variables. Moreover, both experimental groups did not differ in any measured variables.

The effectiveness of both types of web-based intervention was also confirmed in within-group comparisons (Day 0 vs Day 16 and Day 0 vs Day 76). There was a significant increase in emotion regulation and mindful eating, as well as a decrease in emotional eating, uncontrolled eating, negative appreciation of body size, lack of familiarity with one’s body, and the experiencing of negative emotions in both experimental groups (EG I, EG II). However, no significant changes were observed in the abovementioned variables between the measurement on Day 16 and 76.

Two months after completion of the 15-day web-based psychoeducation, no statistically significant reduction for BMI was observed in either experimental group. The significant results of the post-hoc multiple comparisons are presented in figures here.

## Discussion

The effectiveness of both types of web-based psychoeducation is confirmed. Under the influence of psychoeducation, an improvement in functioning in the regulation of emotions, eating behaviors, and body image is observed. Only in reference to BMI is no statistically significant reduction observed in either experimental group.

Our findings confirmed that both types of web-based psychoeducation led to statistically significant changes in emotion regulation on Day 16 and Day 76. In addition, their effectiveness was comparable in emotional regulation. All improvements continued for at least 2 months after the end of the intervention. It can be concluded that both types of web-based psychoeducation were effective in adaptive coping with emotions (the explanation can be found here).

Furthermore, only EG I was characterized by a greater reduction in emotional eating/uncontrolled eating compared to CG on Day 16. However, 2 months later, in both experimental groups (EG I, EG II), significant differences in emotional eating were noted compared with CG. This showed that the trajectory for reducing emotional eating looked slightly different in both types of intervention. Ultimately, participation in both types of intervention resulted in similar changes.

Only the first experimental group (EG I) achieved a higher improvement in reducing negative appreciation of body size both at Day 16 and Day 76 compared to the control group. As for emotion regulation, the type of intervention was not a differentiating factor, while in the area of the abovementioned variable related to body image, the first type was only effective than the second. These results may have been associated with one of the skills developed in the module of emotion regulation. It is worth emphasizing that the ability to differentiate between specific emotions was developed only in EG I (and not EG II). Thus, it may be the case that participants transferred the strategy of designing specific emotional experiences to relationships with their own bodies. Their activity related to emotions in respect of their own body would be based on the following steps: (1) identification of emotional triggers (e.g., social exposure), (2) cognitive assessment of trigger and cognitive restructuring, (3) behavioral experiments, (3) the acceptance of emotions, (4) modulating responses triggered by emotions, (5) implementation of behaviors causing deliberately selected emotions. These steps are consistent with the information presented in the book “*Cognitive Behavior Therapy and Eating Disorders*” (Fairburn 2013) in relation to triggers of maladaptive behaviors related to body and eating.

In terms of mindful eating, lack of familiarity with one’s body, and negative emotions, the web-based psychoeducation did not result in any significant changes—no differences between EG I, EG II, and CG on Day 16 and on Day 76. Moreover, these variables were at a similar level in both experimental groups. This means that both types of intervention did not lead to such strong changes in the abovementioned context, so it was possible to obtain statistically significant differences between the groups (possible explanations can be found here).

It has not been confirmed that the web-based psychoeducation in EG I was the most effective in weight reduction. This may be due to the fact that too short a period of time (only 2 months) from the end of the intervention was considered, because some studies show that the time required to affect a healthy weight change is longer (Alberts et al. 2012; Kristeller et al. 2013; Smart et al. 2015). The idea of mindful eating is contrary to the majority of methods used by people to achieve weight loss in the short term (e.g., dietary restrictions) (Kristeller and Lieberstein 2016; Wnuk and Du 2017). The primary goal of mindful eating is to increase contact with one’s body, thereby reducing emotional and restrictive eating (Kristeller and Wolever 2011; Smart et al. 2015). Only these changes in the long term can lead to weight reduction and prevention of the yo-yo effect (Kristeller and Wolever 2011; Smart et al. 2015).

In EG I and EG II, the level of emotional regulation has been increased as evidenced by statistically significant differences between measurements (Day 0 vs Day 16, Day 0 vs Day 76). However, no further changes in the abovementioned field could be observed—outcomes on Day 16 not differing significantly from Day 76. A similar result in the measurements on Day 16 and Day 76 was observed for all dependent variables in EG I and EG II (except for BMI). Perhaps there was a similar trajectory for the development of changes in all dependent variables, or changes in the one variable affected the level of other variables (e.g., an increase in emotion regulation may have promoted more mindful and healthy eating and a more positive relationship with one’s own body). These assumptions were consistent with the results by Kidwell et al. (2015). These authors have shown that changes in the adaptive regulation of emotions were associated with the development of more adaptive eating habits. Also, the other results of the studies (Bush et al. 2014) provided evidence that the use of mindfulness techniques supported a reduction in negative experience of one’s own body.

In CG, almost all variables remained unchanged on Day 16 and Day 76. A statistically significant decrease in BMI was only observed in this group. A possible explanation may have been the use of restrictive methods of weight loss by participants, a common phenomenon in people with excess body weight (Johns et al. 2014). These behaviors usually lead to rapid weight loss followed by weight gain (the yo-yo effect), especially if people use many dietary restrictions (Osborn et al. 2011; Palm et al. 2017). We can also assume that asking individuals to report their weight could impact on self-monitoring of body weight.

Another factor that could have affected the results was the disproportion between overweight and obese women in the groups (EG I, EG II—more overweight women than obese; CG—a reverse trend). In the future, it will be necessary to evaluate the effectiveness of interventions separately for both groups. It should also be noted that 16.18% of the women had a physical illness. This may have inhibited weight loss, despite the high effectiveness of psychological training (e.g., Bliddal et al. 2014; Green et al. 2015). Moreover, other factors that could have affected the results were those associated with participants’ age and their hormones and metabolic functioning. The reason for choosing premenopausal women was to increase the homogeneity of the group. Mention has been made above of the impact of hormones and metabolic functioning and of differences between the two groups (premenopausal and postmenopausal). Given that the participants are women up to 48 years of age, there are a significant number of these in the premenopausal stage. We should keep in mind the fact that there are also significant hormonal changes during this stage. Therefore, when analyzing our results, it is important to remember this limitation. Additionally, in subsequent studies seeking to gain better insight into how women function, one should ask about the symptoms of menopause (including changes in menses [e.g., cessation of menses or unusually light or heavy periods], hot flushes, and reduced sex drive).

Our study also had the following limitations: (a) only self-description questionnaires to measure variables; (b) some questionnaires did not have Polish validation; (c) use of questionnaires in the online version; (d) taking into account only the negative aspects of body image; (e) lack of motivational training and development of skills in the implementation of intentions; (f) recruiting participants only among volunteers; (g) no consideration of personality and temperamental traits, any depressive or eating disorder symptoms; (h) unspecified level of education and physical activity; (i) no double randomization; (j) the use of a wait list control group. The abovementioned limitations may have been a source of heterogeneity of the group and may have had a negative influence on the validity of the experimental study by increasing within-group variation (Brzeziński and Zakrzewska 2016). Moreover, one of our study’s limitations is that body weight data is not available immediately after the end of the intervention. Some articles suggest that interventions result in meaningful weight loss at the beginning of the intervention, and weight gain a few months after the end of the intervention (e.g., Osborn et al. 2011). Therefore, weight that participants may have lost during the online intervention could have been regained during the follow-up period. Other conclusions can be found here.

To sum up, although the effectiveness of web-based intervention in relation to other studies (e.g., Daly et al. 2016; Mason et al. 2016; Washington et al. 2017; Wnuk et al. 2018; Wnuk and Du 2017) remained satisfactory, future studies will need to make the abovementioned changes. The most important next step would be a longer-term trial to examine whether longer-term trial results in weight loss in overweight and obese individuals. Moreover, in future studies, it should be verified whether our intervention can be used to psychoeducation women both in the psychologist’s office and in interdisciplinary teams. Because there was an extensive research done showing that conducting the online intervention is as effective as the traditional therapy model and introduction of web-based interventions to the traditional treatment was effective as well (Andersson and Titov 2014; Andersson and Hedman 2013; Andersson et al. 2013; Mohr et al. 2010).

## Conclusions

Our psychoeducation can be used as a method to help improve functioning in women with excess body weight, especially those who are diagnosed with the need for mental health intervention. It can be assumed that changes in the field of emotional functioning, eating behaviors, and body image may be important in effective weight management (e.g., Montesi et al. 2016; Teixeira et al. 2015). This may be due to the fact that the improvement in mental well-being will also contribute to an increase in motivation and the level of self-control (Teixeira et al. 2015).

Our eHealth intervention may be particularly useful for people who live far away from obesity treatment centers and are unable to benefit from psychological help in their area. Moreover, a web-based intervention may be a good solution in a situation where direct access to the intervention is interrupted or limited, for example due to the COVID-19 pandemic.

However, it should be remembered that, apart from facilitators, there are also some barriers to implementing the results of our study, which include, among others (a) lack of experience and knowledge of eHealth interventions; (b) lack of necessary devices and technical capacities; (c) lack of permanent access to the Internet; (d) concerns about confidentiality, security, and anonymity; (e) the time-consuming nature of intervention and workload; (f) independently maintaining motivation during the intervention; and (g) lack of face-to-face communication.
